# Seasonal Predictability of the East Atlantic Pattern from Sea Surface Temperatures

**DOI:** 10.1371/journal.pone.0086439

**Published:** 2014-01-22

**Authors:** Isabel Iglesias, María N. Lorenzo, Juan J. Taboada

**Affiliations:** 1 Grupo de Física de la Atmósfera y del Océano. Facultad de Ciencias. Universidad de Vigo, Ourense, Spain; 2 Centro Interdisciplinar de Investigação Marinha e Ambiental, Universidade do Porto, Porto, Portugal; 3 Meteogalicia. Xunta de Galicia. Santiago de Compostela, Spain; University of Aveiro, Portugal

## Abstract

This study analyzes the influence of sea surface temperatures (SSTs) on the second mode of atmospheric variability in the north Atlantic/European sector, namely the East-Atlantic (EA) pattern, for the period 1950–2012. For this purpose, lead-lag relationships between SSTs and the EA pattern, ranging from 0 to 3 seasons, were assessed. As a main result, anomalies of the EA pattern in boreal summer and autumn are significantly related to SST anomalies in the Indo-Pacific Ocean during the preceding seasons. A statistical forecasting scheme based on multiple linear regression was used to hindcast the EA-anomalies with a lead-time of 1 to 2 months. The results of a one-year-out cross-validation approach indicate that the phases of the EA in summer and autumn can be properly hindcast.

## Introduction

Seasonal forecasts are potentially of great benefit for a wide range of socio-economic activities such as agriculture [1], health [2], energy [3], [4] or finance [5]. However, the corresponding forecasting systems are known to have limited skill in the mid-latitudes and any improvement in this field would be of great interest [6].

Since the de-correlation time (or memory) of the tropospheric circulation in the mid-latitudes is limited to about 2 weeks at the utmost, slowly varying boundary systems like sea-surface temperatures [7]–[14], soil-moisture [15], sea-ice [16] and snow cover [17]–[19] are potential sources of seasonal predictability since they 1) are more persistent than the tropospheric circulation and 2) are coupled to the latter, making it potentially predictable.

The present study assesses the lead-lag relationships between SSTs around the entire globe and the extratropical circulation in the North-Atlantic/European sector [11], [20]–[22]. In contrast to previous studies [23], [24], the focus is not put on the predictability of the north Atlantic Oscillation [25], [26], but on the second mode of inter-annual variability of the tropospheric circulation in that area, namely the East Atlantic (EA) pattern [27], [28]. Particularly in southern Europe, the EA pattern is at least as important as the NAO for explaining inter-annual variations of sensible climate variables such as air temperatures, sea-surface temperatures, precipitation and wind [29]–[36], which in turn affect regional- to local-scale ecosystems [37]–[39]. Hence, the predictability of the EA-index is of considerable interest for the development of seasonal forecasting schemes and their applications [6].

The present study is outlined as follows: The applied data sets and the methodology are described in Section 2, the results are presented in Section 3 and a general discussion, including possible dynamical pathways for the detected empirical relationships, is given in Section 4, which also provides the concluding remarks.

## Data and Methods

Serving as predictor variables, the extended reconstructed sea surface temperature (ERSST) dataset version 3 is used in the present study [40]. The data were retrieved from http://www.esrl.noaa.gov/psd/data/gridded/data.noaa.ersst.html and are provided as monthly anomalies on a regular grid of 2 × 2 degrees.

As predictand variable, the EA-index provided by the Climate Prediction Center (http://www.cpc.noaa.gov/data/teledoc/nao.shtml) is used. This index is the Principal Component time series of the second EOF obtained from Rotated Principal Component Analysis, calculated upon monthly anomalies of the geopotential at 500 hPa in the north Atlantic/European sector (20°N–90°N) [27].

For both the predictor and predictand variables, seasonal averages were calculated upon the monthly values. January-to-March is referred to as Winter, April-to-June as Spring, July-to-September as Summer and October-to-November as Autumn. The time period under study is 1950–2012 (n = 63). To eliminate the long-term trend, all series used are linearly detrended and normalized by the corresponding standard deviation prior to the statistical analysis.

Seasonal lags are used in the correlation analyses, e.g. “lag 1″ refers to the correlation between wintertime-mean SST anomalies and the springtime-mean EA index.

The methodology used in this work is the same one used by Philips and McGregor [41] and Lorenzo et al. 2010 [14]. The Pearson correlation coefficient is applied to measure the linear association between the SST at each grid-box of a spatial domain covering the entire globe and the EA index. The significance of the coefficients is assessed by a two-sided Student’s t-test. To additionally take into account that positive serial correlation, e.g. caused by SST-anomaly re-emergence [42], might artificially lower the p-values [43], the latter are optionally calculated upon the effective sample size (see equation 31 in [44] for the formula used to calculate the latter). Since this procedure yielded similar results than applying the standard t-test, which neglects the effect of serial correlation on the p-value, it is reasonable to assume that the time series applied here are temporally independent (see additional material for review).

Since the t-test is applied thousands of times in the present study, significant correlation coefficients are expected to arise by chance for a certain fraction of grid-boxes. For instance, if the local test level is set to 5% and the spatial autocorrelation of the SST time series is assumed to be zero (which is not the case), false rejections of the null hypothesis (type-one errors) are expected to occur in 5% of all test cases.

Therefore, in the present study, the field significance test described in [45] is applied to calculate the fraction of significant correlation coefficients arising by chance, which takes into account the spatial autocorrelation of the SSTs. For this purpose, the EA-index time series was replaced by random Gaussian noise generated from a normal distribution whose mean and variance is identical to the observed time series of the EA-index. The resulting percentage of significant correlations (arising from chance) is saved and the process is repeated 11074 times. The 90^th^ percentile of the resulting sample is then taken as the critical value above which the percentage of significant correlations obtained from the EA-index time series is globally significant at a test level of 10%. This critical value was found to be approximately 15%.

In case global/field significance is obtained, the following procedure is applied: First, those ocean areas where the SST-EA link is locally significant at a test-level of 10% for both the lag-1 and lag-2 correlations are identified. Within these areas having a significant predictive potential for both lags, a maximum of 3 clusters of highest correlations are identified and, for each cluster, the spatial mean SST is calculated for each timestep/season. The resulting time series are then used as predictor variables in a multiple linear regression model. Note that a maximum of 3 clusters/predictors is used in order to limit the parameters of the regression model. To additionally avoid a potential overfit [43], the statistical models are validated in a one-year cross-validation framework [46], i.e. n-1 predictor-predictand pairs are used to obtain the regression equation, which is then used to predict the withheld predictand value. This process is repeated for each predictor-predictand pair, thereby obtaining a hindcast EA-index, which is finally validated against its observed counterpart by using the Pearson correlation coefficient.

Following [47], [48], a multi-category contingency table was used to verify the hindcast EA time series which is categorized into positive (+), neutral and negative phase (−) values using a threshold value of ±0.5 standard deviations from the mean for defining the three categories. The attention will be mainly focused in the positive and negative phases of EA.

## Results

**Figure 1:**
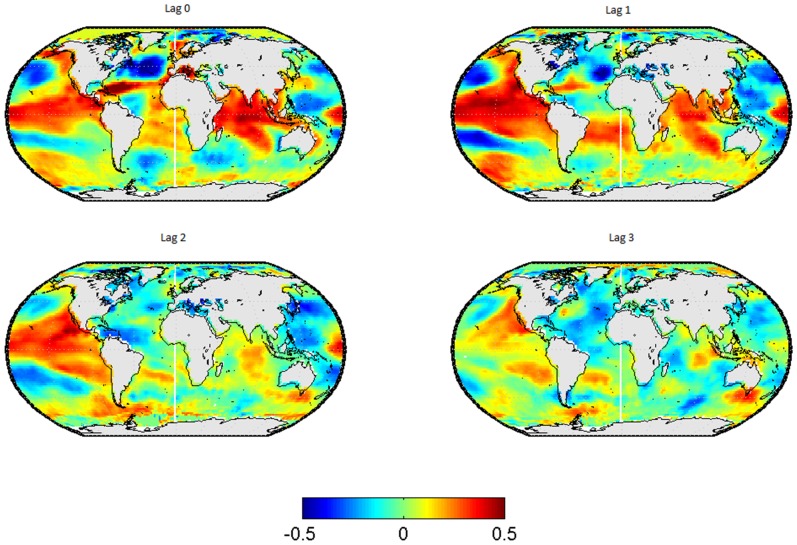
Correlation map between SSTs and the EA pattern in winter. Each subplot represents a different lag, from left to right and from up to down the lag go since 0 to 3.

**Figure 2:**
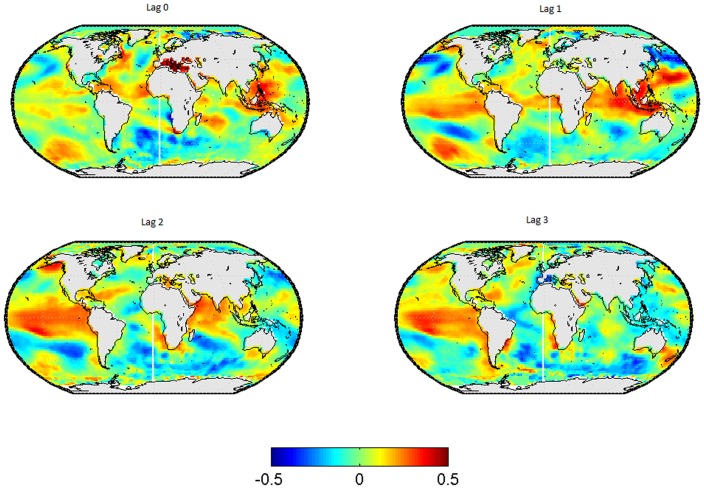
Correlation map between SSTs and the EA-pattern in spring. Each subplot represents a different lag, from left to right and from up to down the lag go since 0 to 3.

**Figure 3:**
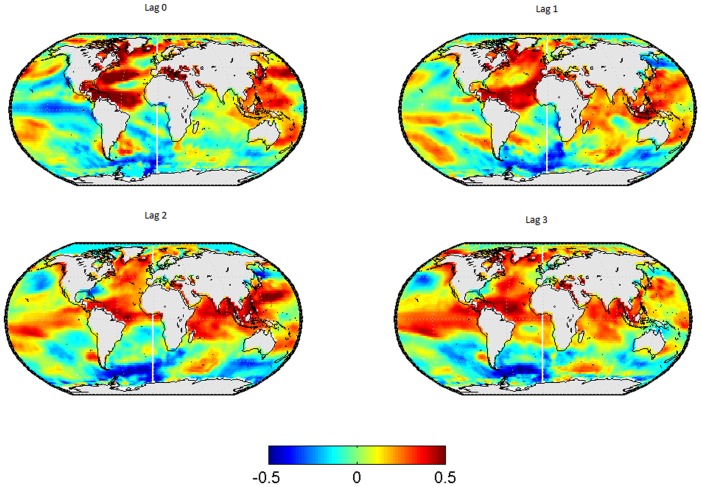
Correlation map between SSTs and the EA-pattern in summer. Each subplot represents a different lag, from left to right and from up to down the lag go since 0 to 3.

**Figure 4:**
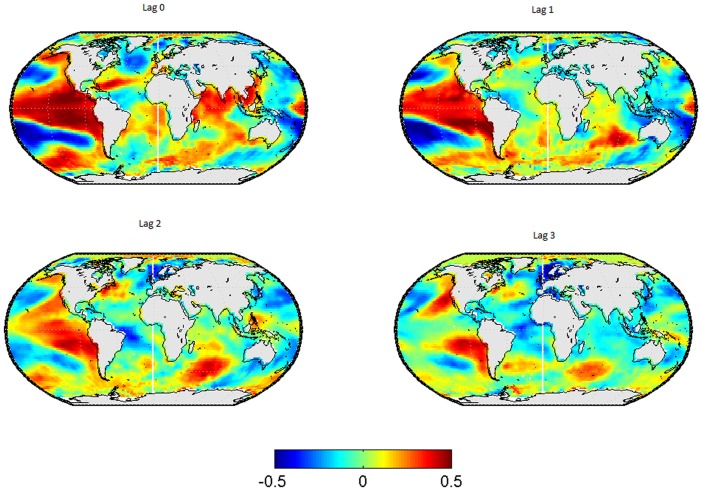
Correlation map between SSTs and the EA-pattern in autumn. Each subplot represents a different lag, from left to right and from up to down the lag go since 0 to 3.

In Figure 1, the lead-lag relationships between SSTs in and the wintertime-mean EA-Index are shown for the SSTs leading by 0 to 3 seasons. The corresponding results for the spring-, summer- and autumn-mean EA-index are displayed in Figures 2 to 4 respectively.

A strong positive correlation between the EA-index in winter and the SSTs in the tropical Pacificand Indian Ocean is observed, whereas the corresponding correlation with the north Atlantic SSTs is predominantly negative (see Figure 1). Both relationships weaken if the lead-time is increased.

If the spring-mean EA-index is used instead of the winter-mean, the magnitude of the correlation coefficients in the tropical Pacific is larger for lag 1, 2 and 3 than for lag 0, indicating that the EA-index is clearly led by preceding SST anomalies (see Figure 2).

For the EA pattern in summer (see Figure 3), areas of significant correlations re-appear over the north Atlantic, as was the case for the EA in winter. However, in contrast to the latter, the relationship is predominantly positive for the EA in summer (compare Figure 3 to Figure 1). At lag 0 and 1, the pattern of significant positive correlations covers both the Tropical- and the north Atlantic Ocean, resembling the well-known tripole- and horseshoe patterns documented in previous studies. For lags 2 and 3, the strength of the relationship is more pronounced in the tropical than in the extra-tropical Atlantic, which confirms the findings of [49] who suggested that SSTs in the north Atlantic are led by SSTs in the tropical Atlantic via an “atmospheric bridge”.

When considering the autumn-mean EA pattern as predictand variable (see Figure 4), large areas of significant correlations are again found over the tropical Pacific and Indian Ocean at lag 0. At longer lags, however, significant correlations are yielded over the tropical Pacific Ocean only.

**Figure 5:**
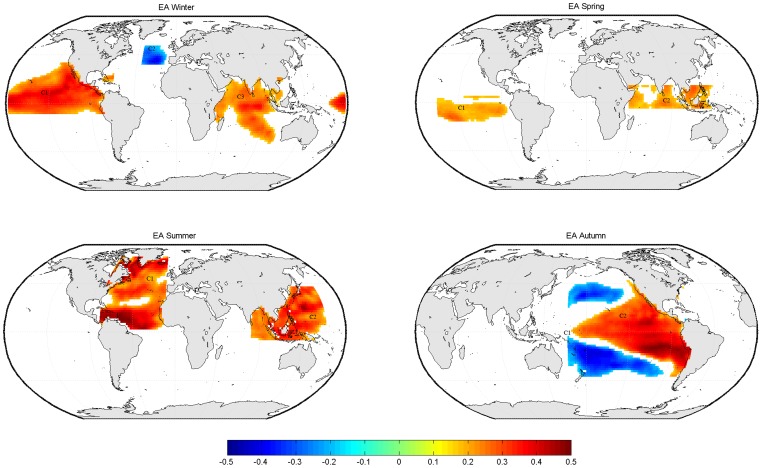
Locations of the clusters used for predicting the EA-pattern in each season.

Figure 5a displays the 3 SST clusters yielding highest correlations with the EA-pattern in winter at both, lag 1 and 2 (seasons). These clusters are located in the tropical Pacific and Indian Ocean (positive relationship), as well in the mid-latitudinal eastern north Atlantic (negative relationship), the latter region being in qualitative agreement with the results of [50]. In comparison with the other seasons, the cluster for the EA in spring are less pronounced (see Figure 5b). The clusters for the EA in summer (see Figure 5c) are located over the north Atlantic Ocean, forming a horseshoe pattern, and over the western Tropical Pacific, also covering the entire Malay Archipelago. The relationship is positive at any grid-box.

Finally, the clusters obtained for the EA in autumn (see Figure 5d) resemble an El Niño-like pattern, with positive correlations over the central to eastern tropical Pacific, flanked by negative ones over the central-north and central-south Pacific Ocean.

For each of the SST clusters displayed in Figure 5, the spatial-average time series was calculated for a lead time of 1 and 2 seasons, thereby obtaining a maximum of 6 predictor variables (3 for each lag) entering the regression model.

**Figure 6:**
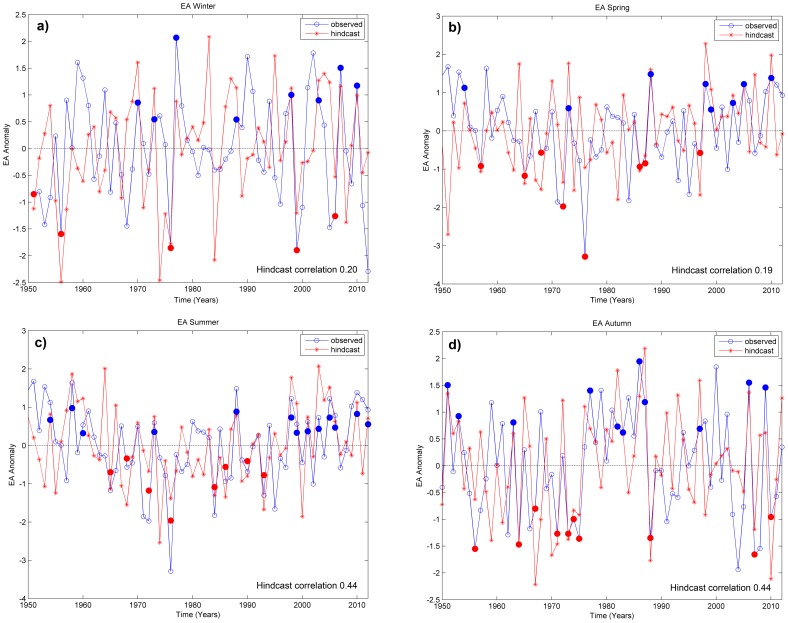
Time series of observed (blue circles) and hincast (red asterisk) EA-Index for (a) winter, (b) spring, (c) summer and (d) autumn. Successful hindcasts of the positive and negative phases of the EA are marked by filled circles. Note that the hindcasts are obtained from one-year-out cross-validation.

A visual comparison between the hindcast time series obtained from cross-validation and their corresponding observed time series is provided by Figure 6 for each season of the year. The correlation between hindcasts and observations, hereafter referred to as “hindcast correlation”, is 0.44 for predicting the EA-pattern in summer and autumn, which is significant at a test-level of 1%. Note that the corresponding critical value is 0.32 (using a two sided t-test with 61 degrees of freedom).

The results of the contingency analysis (see Table 1 and Table 2) reveal that the phases of the EA-pattern in summer and autumn are successfully hindcast, (see value of PC in Table 2). Albeit the corresponding percentages for winter and spring are lower. The corresponding false alarm rate (see value of F in table 2) for positive and negative phase are below 30%.

**Table 1:** Contingency Tables using equations of multiple linear regression models to forecast EA phases.

EA WINTER		Observed			
		Positive Phase	Neutral Phase	Negative Phase	Total
**Forecast**	**Positive Phase**	8	6	5	19
	**Neutral Phase**	10	8	9	27
	**Negative Phase**	3	8	5	16
	Total	21	22	19	62
**EA SPRING**		**Observed**			
		**Positive Phase**	**Neutral Phase**	**Negative Phase**	**Total**
**Forecast**	**Positive Phase**	8	4	4	16
	**Neutral Phase**	7	14	6	27
	**Negative Phase**	7	5	8	20
	Total	22	23	18	63
**EA SUMMER**		**Observed**			
		**Positive Phase**	**Neutral Phase**	**Negative Phase**	**Total**
**Forecast**	**Positive Phase**	14	4	2	20
	**Neutral Phase**	4	13	8	25
	**Negative Phase**	4	6	8	18
	Total	22	23	18	63
**EA AUTUMN**		**Observed**			
		**Positive Phase**	**Neutral Phase**	**Negative Phase**	**Total**
**Forecast**	**Positive Phase**	11	7	4	22
	**Neutral Phase**	6	9	8	23
	**Negative Phase**	5	3	10	18
	Total	22	19	22	63

**Table 2:** Verification measures of the Contingency Tables.

	Winter	Spring	Summer	Autumn
**Positive Phase**				
PC	0.61	0.65	0.78	0.65
F	0.27	0.20	0.15	0.27
HSS	0.12	0.18	0.50	0.23
**Neutral Phase**				
PC	0.47	0.65	0.65	0.62
F	0.47	0.32	0.30	0.32
HSS	0.0	0.27	0.26	0.15
**Negative Phase**				
PC	0.60	0.65	0.68	0.68
F	0.26	0.28	0.22	0.20
HSS	0.0	0.17	0.22	0.27

PC = Percentage of correct forecasts; F = False Alarm Rate; HSS = Heidke Skill Score.

## Discussion and Conclusions

The physical rationale linking tropical Pacific SSTs/the ENSO phenomenon to subsequent climate anomalies in the north Atlantic/European sector have been discussed in many previous studies (see e.g. [51] and references therein). One possible explanation is that ENSO is coupled to the stratospheric polar vortex in winter, whose anomalies are known to propagate downward [52], thereby influencing the tropospheric circulation in the north Atlantic/European sector [53]. This dynamical pathway, however, is bounded to the lifetime of the polar vortex, and a detectable influence on the European climate was found for the (late) winter season only [54]. Consequently, the dynamical pathway involving the polar stratospheric vortex cannot explain the statistical links found here for the EA pattern in other seasons.

An alternative physical explanation is provided by the theory that the SSTs in different ocean basins are linked by an “atmospheric bridge” [49]. Following this theory, SST anomalies in the north Atlantic during spring and summer, which are known to be informative predictors of the autumn and winter climate in Europe [11], [20], [55], are led by SST anomalies in the tropical Pacific [56], which is consistent to the findings presented here.

This study has revealed that sea surface temperatures in the tropical Pacific- and Indian Ocean as well as in the north Atlantic are informative statistical predictors for the phase of East Atlantic Pattern in summer and autumn, which is known to be associated with concurrent climate anomalies (as e.g. represented by precipitation and temperature) in southern Europe. Statistical predictions based on multiple linear regression, which are validated in a one-year-out cross-validation framework, reveal that approximately 20% of the inter-annual variability of the EA in summer and autumn can be explained by the above mentioned predictors and that the phase of the EA in these seasons can be correctly hindcast in at least 65% of all cases.
